# Lefty Blocks a Subset of TGFβ Signals by Antagonizing EGF-CFC Coreceptors

**DOI:** 10.1371/journal.pbio.0020030

**Published:** 2004-02-17

**Authors:** Simon K Cheng, Felix Olale, Ali H Brivanlou, Alexander F Schier

**Affiliations:** **1**Developmental Genetics Program, Skirball Institute of Biomolecular Medicineand Department of Cell Biology, New York University School of Medicine, New York, New YorkUnited States of America; **2**Laboratory of Molecular Vertebrate Embryology, The Rockefeller UniversityNew York, New YorkUnited States of America

## Abstract

Members of the EGF-CFC family play essential roles in embryonic development and have been implicated in tumorigenesis. The TGFβ signals Nodal and Vg1/GDF1, but not Activin, require EGF-CFC coreceptors to activate Activin receptors. We report that the TGFβ signaling antagonist Lefty also acts through an EGF-CFC-dependent mechanism. Lefty inhibits Nodal and Vg1 signaling, but not Activin signaling. Lefty genetically interacts with EGF-CFC proteins and competes with Nodal for binding to these coreceptors. Chimeras between Activin and Nodal or Vg1 identify a 14 amino acid region that confers independence from EGF-CFC coreceptors and resistance to Lefty. These results indicate that coreceptors are targets for both TGFβ agonists and antagonists and suggest that subtle sequence variations in TGFβ signals result in greater ligand diversity.

## Abstract 2

TGFβ family members and their receptors are involved in setting up the left-right body axis early in development. This article clarifies the role of Lefty and elucidates the molecular basis for signaling diversity between the family members

## Introduction

The analysis of whole-genome sequences has revealed that most signaling systems consist of multiple ligands that converge on a relatively small set of receptors and pathway-specific transcription factors. In the case of human transforming growth factor-β (TGFβ) signaling, 42 TGFβs converge on seven type I receptors, five type II receptors, and two classes of Smad signal transducers (reviewed in Shi and Massague 2003). This convergence has raised the question of how ligand diversity and signaling specificity among different signals can be achieved. If different TGFβs activate the same receptors, it is unclear how these ligands can vary in their function (diversity) or how a given signal can have a unique role (specificity). Biochemical studies have suggested that ligand diversity can be attained by differential stability and receptor affinity, leading to differences in signaling strength (reviewed in Piek et al. 1999; Shi and Massague 2003). An additional source of ligand variability stems from differential ligand movement through a field of cells, rendering related signals either short- or long-range (Chen and Schier 2001). Finally, specificity and diversity can also be determined by ligand-specific cofactors or inhibitors (Piek et al. 1999; Shi and Massague 2003). A prominent example involves epidermal growth factor–Cripto/FRL-1/Cryptic (EGF-CFC) coreceptors and the TGFβs Activin, Nodal, and Vg1/GDF1 (growth and differentiation factor-1). In this case, differential dependence on a coreceptor leads to ligand diversity and signaling specificity (reviewed in Schier 2003).

Members of the Nodal, Activin, and Vg1/GDF1 subfamilies display similar activities and are potent mesendoderm inducers in vertebrates (reviewed in Schier and Shen 2000). Genetic and biochemical studies have shown that EGF-CFC proteins are essential for signaling by Nodal and Vg1/GDF1 (Gritsman et al. 1999; Reissmann et al. 2001; Yeo and Whitman 2001; Bianco et al. 2002; Sakuma et al. 2002; Yan et al. 2002; Cheng et al. 2003). EGF-CFC proteins are extracellular glycosylphosphatidylinositol (GPI)-linked factors and include One-eyed pinhead (Oep) in zebrafish and mammalian Cripto and Cryptic (reviewed in Shen and Schier 2000; Minchiotti et al. 2002; Schier 2003). Genetic studies in zebrafish and mouse have shown that EGF-CFC proteins and Nodal are required for mesoderm and endoderm induction (Conlon et al. 1991, 1994; Zhou et al. 1993; Ding et al. 1998; Feldman et al. 1998; Gritsman et al. 1999). For example, zebrafish embryos lacking both the maternal and zygotic contribution of Oep (MZ*oep*) lack all endoderm and most mesoderm, similar to the double mutants for the zebrafish Nodal-related genes *cyclops* and *squint* (*sqt*) (Feldman et al. 1998; Gritsman et al. 1999). Moreover, Nodal and Vg1/GDF1 are inactive in MZ*oep* mutants (Gritsman et al. 1999; Cheng et al. 2003). During later stages of development, Oep, Cryptic, Nodal, and GDF1 are required for proper left–right axis formation (Gaio et al. 1999; Yan et al. 1999; Bamford et al. 2000; Rankin et al. 2000; Brennan et al. 2002; Long et al. 2003).

The EGF-CFC protein Cripto is highly overexpressed in human epithelial cancers, such as breast and colon carcinomas (reviewed in Salomon et al. 2000), and has been implicated in tumorigenesis (Ciardiello et al. 1991, 1994; Baldassarre et al. 1996; De Luca et al. 2000; Salomon et al. 2000; Adkins et al. 2003). The mechanism by which Cripto mediates tumorigenesis is not well understood. Several possibilities include mediating Nodal/GDF1 signaling (Gritsman et al. 1999; Reissmann et al. 2001; Yeo and Whitman 2001; Bianco et al. 2002; Sakuma et al. 2002; Yan et al. 2002; Cheng et al. 2003), antagonizing Activin signaling (Adkins et al. 2003; Gray et al. 2003), or activating Akt and mitogen-activated protein kinase (MAPK) pathways independently of the TGFβ signals and Activin receptors (Ebert et al. 1999; Bianco et al. 2002, 2003). Whatever the molecular mechanism of Cripto activity, inhibition of Cripto by antisense or antibody blockade can inhibit tumor cell proliferation in vitro and in vivo (Ciardiello et al. 1994; Baldassarre et al. 1996; De Luca et al. 2000; Adkins et al. 2003).

Biochemically, EGF-CFC proteins can act as coreceptors for Nodal and Vg1/GDF1 to bind and activate the type I Activin receptor Alk4 and the type II Activin receptor ActRIIB (Reissmann et al. 2001; Yeo and Whitman 2001; Sakuma et al. 2002; Yan et al. 2002; Bianco et al. 2002; Cheng et al. 2003). In the absence of EGF-CFC proteins, these TGFβs cannot form a complex with Activin receptors. Strikingly, Activin utilizes the same receptors as Nodal and Vg1/GDF1, but does not require EGF-CFC coreceptors (Mathews and Vale 1991; Attisano et al. 1992, 1996; Hemmati-Brivanlou and Melton 1992; Mathews et al. 1992; ten Dijke et al. 1994; Chang et al. 1997). For instance, Activin can signal in MZ*oep* mutants (Gritsman et al. 1999). This ligand diversity between Activin and Nodal or Vg1/GDF1 raises the question of which sequences confer coreceptor dependence or independence. Activin, Nodal, and Vg1/GDF1 are highly related and are thought to acquire very similar folds. Like other TGFβ ligands, Activin has four major structural features: a β-stranded Finger 1, an α-helical Heel, a β-stranded Finger 2, and three conserved disulfide bonds that form a cysteine knot (Shi and Massague 2003; Thompson et al. 2003). Sequence comparisons indicate that the highest divergence among Activin, Nodal, and Vg1/GDF1 is in the N-terminal segment of Finger 1, the central α-helix, and the loop of Finger 2 with approximately 10%, approximately 15%, and approximately 25% sequence identity, respectively. These regions are potential candidates to determine the specificity of receptor–coreceptor–ligand interactions.

In addition to coreceptors, antagonists represent another class of extracellular factors that control ligand access to receptors (reviewed in Piek et al. 1999; Freeman 2000; De Robertis et al. 2001; Shi and Massague 2003). For example, the divergent TGFβ class of Lefty proteins antagonizes Nodal signaling (reviewed in Hamada et al. 2002; Schier 2003). Unlike other TGFβs, Lefty proteins may function as monomers due to the lack of a cysteine residue involved in dimerization (Meno et al. 1996; Thisse and Thisse 1999; Sakuma et al. 2002). Lefty overexpression in zebrafish induces phenotypes identical to *cyclops;sqt* double mutants and MZ*oep* mutants (Bisgrove et al. 1999; Meno et al. 1999; Thisse and Thisse 1999). Furthermore, the loss of Lefty activity leads to enhanced Nodal signaling during mesoderm induction and left–right axis determination (Meno et al. 1999, 2001; Agathon et al. 2001; Branford and Yost 2002; Chen and Schier 2002; Feldman et al. 2002). Although it has not been determined whether Lefty directly blocks Vg1/GDF1 signaling (Branford et al. 2000), it has been proposed that Lefty inhibits signaling by Activin. Misexpression of Activin or ActRIIB can overcome the inhibitory effects of Lefty (Meno et al. 1999; Thisse and Thisse 1999; Cheng et al. 2000; Tanegashima et al. 2000; Sakuma et al. 2002). Hence, some members of the Lefty family have been called Antivins for their anti-Activin properties (Thisse and Thisse 1999; Cheng et al. 2000; Ishimaru et al. 2000; Tanegashima et al. 2000). However, it has been elusive how Lefty functions at the molecular level.

Here we present genetic and biochemical studies in zebrafish and *Xenopus* that indicate that Lefty is an in vivo antagonist of EGF-CFC coreceptors. We find that Lefty can antagonize signaling by the coreceptor-dependent ligands Nodal and Vg1/GDF1, but not Activin. Lefty genetically interacts with Cripto and Oep and competes with Nodal for binding to Cripto, representing a novel mechanism for antagonizing TGFβ signaling. We identify a short region in Finger 2 of Activin, Nodal, and Vg1 that determines EGF-CFC coreceptor-dependent or coreceptor-independent signaling and governs susceptibility to Lefty. These results indicate that subtle sequence variations in TGFβ ligands can dramatically expand signaling diversity by determining interactions with coreceptors and their antagonists.

## Results

### Lefty Antagonizes Nodal and Vg1 Signaling, but Not Activin Signaling

**Figure 1:**
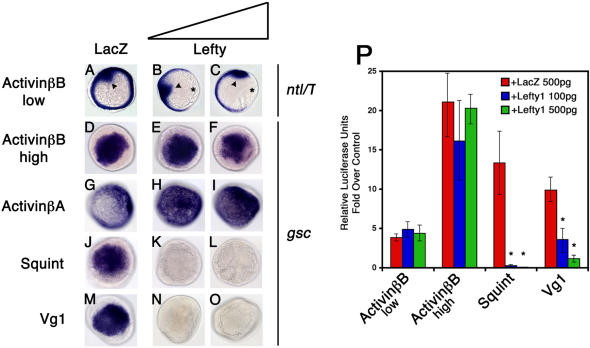
Lefty Antagonizes Nodal and Vg1 Signaling, but Not Activin Signaling, in Zebrafish *ntl* mRNA expression (A–C) and *gsc* mRNA expression (D–O) in wild-type zebrafish embryos at shield stage, animal pole view. Embryos were injected with low levels (1 pg) of *activin βB* mRNA (A–C), high levels (10 pg) of *activin βB* mRNA (D–F), 200 pg of *activin βA* mRNA (G–I), 75 pg of *sqt* mRNA (J–L), or 200 pg of *Vg1* (M–O). Embryos were further double-injected with either 500 pg of *LacZ* mRNA (A, D, G, J, and M), 100 pg of *lefty1* and 400 pg of *LacZ* mRNAs (B, E, H, K, and N), or 500 pg of *lefty1* mRNA (C, F, I, L, and O). Ectopic *ntl* expression (arrowheads) in *activin βB* mRNA-injected embryos was not inhibited by Lefty1 (B and C) when compared with *LacZ* mRNA-coinjected controls (A). Note the dorsal expression of *ntl* (asterisks)—that is, dependent on endogenous Nodal signaling—is inhibited by Lefty1 in these embryos (B and C). Ectopic *gsc* expression in *activin βB* and *activin βA* mRNA-injected embryos was not inhibited by Lefty1 (E and F and H and I, respectively). In contrast, ectopic *gsc* expression in *sqt* and *Vg1* mRNA-injected embryos was inhibited by both levels of Lefty expression (K and L and N and O, respectively). Wild-type embryos (P) were injected with 10 pg (low) and 20 pg (high) of *activin βB*/HA, 75 pg of *sqt*, or 200 pg of *Vg1* mRNA. Embryos were further double-injected with 500 pg of *LacZ* mRNA, 100 pg of *lefty1*, and 400 pg of *LacZ* mRNAs, or 500 pg of *lefty1* mRNA. Smad2 pathway activation was measured by an Activin response element luciferase reporter, A3-luc. Values are folds over wild-type control injected with 500 pg of *LacZ* mRNA and A3-luc reporter. An asterisk indicates a significant difference from the level of activation with ligand and *LacZ* expression alone (Student's *t*-test, *p* < 0.05).

TGFβ ligands that activate Activin receptors can be categorized into two classes. The Activin class activates Activin receptors in an EGF-CFC coreceptor-independent manner, whereas the Nodal and Vg1/GDF1 classes require EGF-CFC proteins for receptor activation (Gritsman et al. 1999; Cheng et al. 2003). To determine whether these classes are also differentially susceptible to inhibition by the TGFβ antagonist Lefty, we coexpressed zebrafish Lefty1 with *Xenopus* ActivinβB, *Xenopus* ActivinβA, Sqt (a zebrafish Nodal-related protein), or zebrafish Vg1 (a chimeric molecule containing the *Xenopus* ActivinβB prodomain fused to the mature domain of zebrafish Vg1) in zebrafish embryos (Smith et al. 1990; Thomsen et al. 1990; van den Eijnden-Van Raaij et al. 1990; Helde and Grunwald 1993; Erter et al. 1998; Feldman et al. 1998; Thisse and Thisse 1999). As a readout for active signaling, we analyzed the ectopic induction of the Nodal downstream gene *goosecoid* (*gsc*). ActivinβB, ActivinβA, Sqt, and Vg1-induced ectopic *gsc* expression in wild-type embryos (Figure 1D, 1G, 1J, and 1M; Gritsman et al. 1999; Cheng et al. 2003). Coexpression of Lefty1 efficiently inhibited *gsc* induction by Sqt (Figure 1K and 1L; Bisgrove et al. 1999; Meno et al. 1999; Thisse et al. 2000) and Vg1 (Figure 1N and 1O), but not ActivinβB or ActivinβA (Figure 1E, 1F, 1H, and 1I). To examine whether Lefty1 can antagonize the induction of a gene that responds to very low levels of Activin signaling, we titrated ActivinβB levels so that *no tail* (*ntl*; also known as *brachyury/T*) expression was only weakly induced (see arrowhead in Figure 1A). The coexpression of Lefty1 did not inhibit *ntl* induction by ActivinβB (Figure 1B and 1C), but inhibited the dorsal margin expression of *ntl* that is dependent on endogenous Nodal signaling (see asterisks in Figure 1B and 1C; Feldman et al. 1998). In a more quantitative assay, we overexpressed Lefty1, ActivinβB, Sqt, and Vg1 in zebrafish embryos in the presence of the luciferase reporter A3-luc, which contains FoxH1/P-Smad2 response elements (Chen et al. 1996). Consistent with the *gsc* and *ntl* induction assays, Sqt and Vg1 signaling, but not ActivinβB signaling, is inhibited by Lefty1 (Figure 1P). These results indicate that Lefty1 efficiently antagonizes Nodal and Vg1/GDF1 signaling, but not Activin signaling.

### EGF-CFC Proteins Genetically Interact with Lefty

**Figure 2:**
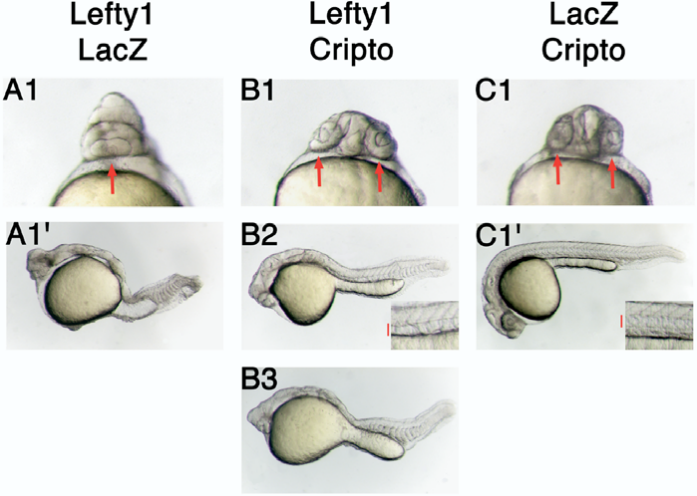
EGF-CFC Coreceptors Genetically Interact with Lefty Live wild-type zebrafish embryos at 30 h postfertilization (hpf). (A1, B1, and C1) Ventral views of the head. (A1′, B2, B3, and C1′) Lateral views, with anterior to the left, dorsal up. (A1, A1′, B1, B2, and B3) Wild-type embryos were injected with 20 pg of *lefty1* mRNA. Embryos were further double-injected with either 200 pg of *LacZ* mRNA (A1 and A1′) or 200 pg of *Cripto* mRNA (B1, B2, and B3). (C1 and C1′) Wild-type embryos injected with 200 pg of *Cripto* mRNA and 20 pg of *LacZ* mRNA. Misexpression of Lefty1 results in cyclopia and other head and trunk mesoderm defects ([A1 and A1′] 32 of 32 embryos had the phenotype shown; arrow shows cyclopia). Coexpression of Cripto with Lefty in embryos leads to rescue of two eyes ([B1] four of 50; arrows show two eyes), notochord ([B2] 20 of 50; inset shows trunk somites and notochord, red bar delineates notochord), and trunk somites ([B3] 50 of 50). Embryos injected with *Cripto* mRNA only show normal wild-type phenotype ([C1 and C1′] 30 of 30; arrow in [C1] shows two normal eyes, and inset in [C1′] shows normal notochord and trunk somites, red bar delineates notochord).

The molecular mechanism of Lefty action has been unresolved. Lefty seems to act upstream of the Activin receptors, as Lefty cannot block signaling from ligand-independent constitutively activated receptors (Thisse and Thisse 1999). Our finding that Lefty blocks Nodal and Vg1 signaling, but not Activin signaling, suggests that Lefty blocks extracellular components specific to the Nodal and Vg1 pathways. The only such factors identified to date are the EGF-CFC coreceptors. We therefore examined whether the EGF-CFC genes zebrafish *oep* and mouse *Cripto* genetically interact with Lefty1. Overexpression of Lefty1 in wild-type zebrafish resulted in embryos lacking head and trunk mesendoderm due to inhibition of endogenous Nodal signaling (Figure 2A1 and 2A′; Bisgrove et al. 1999; Meno et al. 1999; Thisse and Thisse 1999). Coexpression of Cripto or Oep partially suppressed Lefty-induced defects (Figure 2B1–2B3; data not shown), restoring trunk and head mesoderm, including the notochord, and resulting in the separation of the eye field into two eyes. These results indicate an antagonistic relationship between EGF-CFC coreceptors and Leftys.

### Lefty Binds to Cripto, but Not to ActRIIB or Alk4

**Figure 3:**
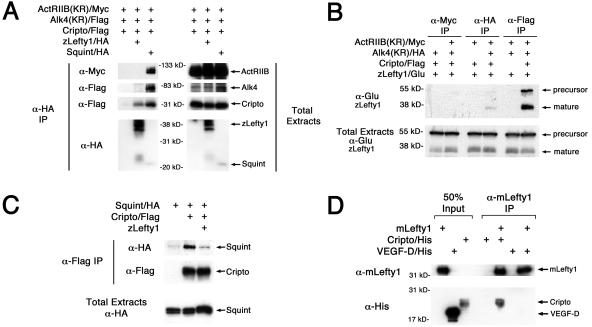
Lefty Binds to Cripto, but Not to the Activin Receptors ActRIIB and Alk4 (A and B) Lefty1 interacts with Cripto. RNAs (1 ng each) encoding ActRIIB(KR)/Myc, Alk4(KR)/Flag, Cripto/Flag, Lefty1/HA, or Sqt/HA were injected into *Xenopus* embryos. After chemical cross-linking, lysates were immunoprecipitated for either Lefty1/HA or Sqt/HA (A) with anti-HA antibody, or ActRIIB(KR)/Myc, Alk4(KR)/HA, Cripto/Flag (B) with, respectively, anti-Myc, anti-HA, or anti-Flag antibodies. Note that Lefty1 specifically interacts with Cripto (A and B), and these Lefty/Cripto complexes do not contain Alk4 (A). Moreover, processed Lefty1 binds much more efficiently to Cripto than full-length Lefty1 precursor (B). In contrast, Sqt can bind to ActRIIB, Alk4, and Cripto (A). The 55 kDa protein marker in (B) is estimated based on molecular weight markers. (C) Lefty1 competes with Nodal for binding to Cripto. RNAs encoding Sqt/HA (1 ng), Cripto/Flag (100 pg), or Lefty1 (2 ng) were injected and anti-Flag antibody was used to immunopreciptate Cripto/Flag. (D) mLefty1 binds directly to Cripto. Purified mouse Lefty1 protein (mLefty1; 10 μg/ml) was mixed with either soluble purified Cripto/His protein (5 μg/ml) or purified control VEGF-D/His protein (5 μg/ml). After chemical cross-linking, mLefty1 was immunoprecipitated with anti-mLefty1 antibody. mLefty1 associates with Cripto, but not with control VEGF-D. Proteins in the coimmunoprecipitates and total extracts were probed in Western blot analysis with the indicated antibodies: ActRIIB(KR)/Myc (kinase-defective receptor, approximately 120 kDa; anti-Myc), Alk4(KR)/Flag (kinase-defective receptor, approximately 70 kDa; anti-Flag), Cripto/Flag (approximately 30 kDa; anti-Flag), Lefty1/HA (mature ligand, approximately 36–40 kDa; anti-HA; Sakuma et al. 2002), Sqt/HA (unprocessed precursor, approximately 55 kDa; mature ligand, approximately 22 kDa; anti-HA), Lefty1/Glu (unprocessed precursor, approximately 55 kDa; mature ligand, approximately 38 kDa; anti-Glu; Sakuma et al. 2002), mLefty1 (mature ligand, approximately 36 kDa, anti-mLefty1; Sakuma et al. 2002), Cripto/His (soluble form, approximately 22–25 kDa; anti-His), and VEGF-D/His (mature ligand, approximately 15–20 kDa; anti-His).

Because Lefty and EGF-CFC proteins interact genetically, we examined whether Lefty interacts biochemically with Cripto and/or ActRIIB/Alk4 receptor complexes. We expressed and immunoprecipitated epitope-tagged ligands (zebrafish Lefty1/HA [hemagglutinin], zebrafish Lefty1/Glu, or zebrafish Sqt/HA), receptors (mouse ActRIIB[KR]/Myc and human Alk4[KR]/Flag), and a coreceptor (mouse Cripto/Flag) in *Xenopus* embryos (Yeo and Whitman 2001; Cheng et al. 2003). Similar to other Nodal-related proteins (Reissmann et al. 2001; Yeo and Whitman 2001; Bianco et al. 2002; Sakuma et al. 2002; Yan et al. 2002), Sqt formed a complex with the type II receptor ActRIIB, type I receptor Alk4, and Cripto (Figure 3A). In contrast, Lefty1 coimmunoprecipitated Cripto, but not ActRIIB or Alk4 (Figure 3A). Since Cripto is bound to Alk4 even in the absence of ligand (Reissmann et al. 2001; Yeo and Whitman 2001; Bianco et al. 2002; Yan et al. 2002), Lefty seemed to disrupt the Cripto–Alk4 interaction. In reverse experiments, Cripto efficiently coimmunoprecipitated Lefty1 (Figure 3B). Since Sqt and Lefty1 can both bind to Cripto (Figure 3C; Reissmann et al. 2001; Yeo and Whitman 2001; Bianco et al. 2002; Sakuma et al. 2002; Yan et al. 2002), these two ligands might compete for binding to Cripto. Indeed, the coexpression of Lefty1 led to decreased interactions between Cripto and Sqt (Figure 3C). To determine whether Cripto can directly interact with Lefty, we immunoprecipitated purified mouse Lefty1 protein (mLefty1) in the presence of either purified mouse Cripto protein or a control cysteine-rich protein, mouse vascular endothelial growth factor-D (VEGF-D). mLefty1 protein directly interacted with Cripto, but not with VEGF-D. Together, these results suggest that Lefty inhibits Nodal signaling by associating with Cripto and blocking it from interacting with Nodal.

### Activin Loop-β8 Region Confers EGF-CFC Coreceptor Independence to Sqt

**Figure 4:**
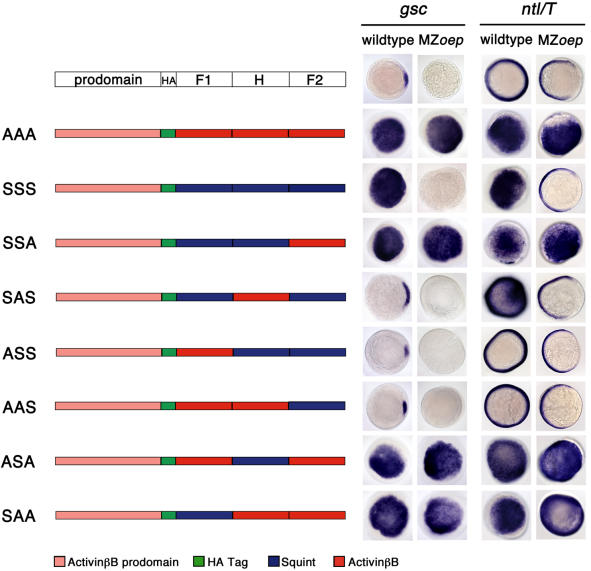
Chimera Analysis to Identify TGFβ Sequence Determinants Conferring EGF-CFC Coreceptor Dependence or Independence Schematic depiction of chimeras of mature ligand domains, Finger 1 (F1), Heel (H), and Finger 2 (F2), between *Xenopus* ActivinβB and zebrafish Sqt. HA indicates an hemagglutinin epitope tag. Schematic is not drawn to scale. The letters in these three-lettered (XXX) chimeras represent the Finger 1, Heel, and Finger 2, respectively. *S* denotes Squint; *A* denotes ActivinβB. Synthetic mRNAs (200 pg) encoding chimeras were injected into wild-type and MZ*oep* embryos. *gsc* and *ntl* mRNA expression is shown at shield stage; animal pole views are dorsal to the right. *gsc* is expressed in the dorsal organizer (shield) in wild-type embryos, but is absent in MZ*oep* mutants. *ntl* is expressed around the entire margin in wild-type embryos, but the dorsal margin expression is lost in MZ*oep* mutants. The presence of the ActivinβB prodomain and epitope tag does not alter the specificity or functionality of wild-type ActivinβB (AAA) or Sqt (SSS). AAA can induce ectopic *gsc* and *ntl* expression in both wild-type and MZ*oep* embryos. In contrast, SSS can induce ectopic *gsc* and *ntl* expression in only wild-type embryos. Similar to ActivinβB, chimeras SSA, SAS, ASA, and SAA can induce ectopic *gsc* and *ntl* expression in both wild-type and MZ*oep* embryos. Chimeras ASS and AAS are inactive in both wild-type and MZ*oep* embryos. Western blot analysis indicated that all chimeric constructs produce stable ligands (data not shown).

**Figure 5:**
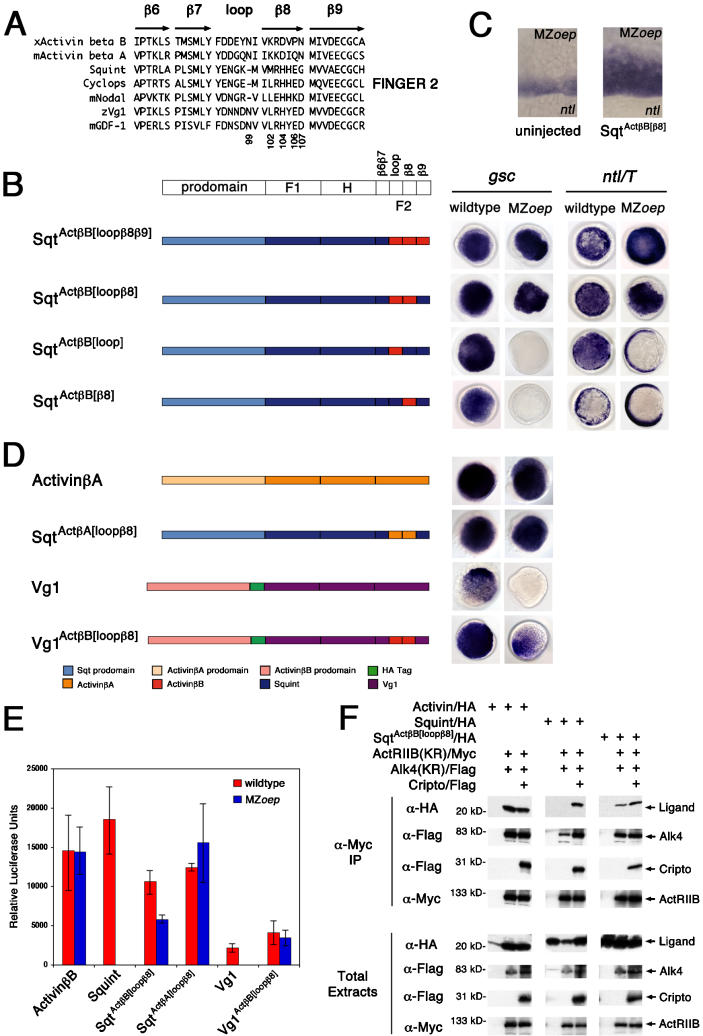
Sequence Determinants Conferring Independence from EGF-CFC Coreceptors (A) Sequence alignment of Finger 2 region of EGF-CFC-dependent and EGF-CFC-independent TGFβ ligands. Location of secondary structure elements, β-sheets (β6–β9) and loop, are shown (Kirsch et al. 2000). Residue numbering is from mouse ActivinβA. (B–E) Synthetic mRNAs (200 pg) encoding chimeras of Finger 2 subregions between *Xenopus* ActivinβB or ActivinβA and zebrafish Sqt or Vg1 were injected into wild-type and MZ*oep* embryos. Schematic is not drawn to scale. *gsc* and *ntl* mRNA expression is at shield stage; animal pole views are dorsal to the right. (B) Sqt^Act^ ^β^ ^B[loop^ ^β^ ^8^ ^β^ ^9]^ and Sqt^Act^ ^β^ ^B[loop^ ^β^ ^8]^ can induce *gsc* and *ntl* expression in both wild-type and MZ*oep* embryos. (C) Sqt^Act^ ^β^ ^B[^ ^β^ ^8]^ can weakly expand *ntl* expression in MZ*oep* mutants. *ntl* mRNA expression in MZ*oep* mutants is at shield stage; lateral view. (D) Other TGFβs conform to loop-β8 EGF-CFC-independent determinant. Note that *Xenopus* ActivinβA can induce ectopic *gsc* in both wild-type and MZ*oep* embryos. In contrast, Vg1 can only induce *gsc* in wild-type embryos. Similar to Activins, chimeric Sqt^Act^ ^β^ ^A[loop^ ^β^ ^8]^ and Vg1^Act^ ^β^ ^B[loop^ ^β^ ^8]^ can induce ectopic *gsc* in both wild-type and MZ*oep* embryos. (E) Wild-type and MZoep embryos were injected with 5 pg of *activin βB*, 100 pg of *sqt*, 100 pg of *Vg1*, 125 pg of *Sqt^ActβB[loopβ8]^*, 250 pg of *Sqt^ActβA[loopβ8]^*, or 100 pg of *Vg1^ActβB[loopβ8]^* mRNA. Smad2 pathway activation was measured by an Activin response element luciferase reporter, A3-luc. Luciferase units are relative to wild-type or MZ*oep* control injected with the A3-luc reporter alone. (F) Sqt^Act^ ^β^ ^B[loop^ ^β^ ^8]^ can bind to ActRIIB and Alk4 in the absence of EGF-CFC coreceptors. RNAs (1 ng each) encoding ActRIIB(KR)/Myc, Alk4(KR)/Flag, Cripto/Flag, ActivinβB/HA, Sqt/HA, or Sqt^Act^ ^β^ ^B[loop^ ^β^ ^8]^/HA were injected into *Xenopus* embryos. Proteins in the coimmunoprecipitates and total extracts were probed in Western blot analysis with the indicated antibodies: ActRIIB(KR)/Myc (approximately 120 kDa; anti-Myc), Alk4(KR)/Flag (approximately 70 kDa; anti-Flag), Cripto/Flag (approximately 30 kDa; anti-Flag), ActivinβB/HA (mature ligand, approximately 16 kDa; anti-HA), Sqt/HA (mature ligand, approximately 22 kDa; anti-HA), and Sqt^Act^ ^β^ ^B[loop^ ^β^ ^8]^/HA (mature ligand, approximately 22 kDa; anti-HA).

The finding that TGFβ ligands that activate Activin receptors can be grouped into a EGF-CFC coreceptor-dependent class that is susceptible to inhibition by Lefty (Nodal and Vg1) and a class that is independent of EGF-CFC proteins and resistant to Lefty (Activin) prompted us to examine which sequences underlie this ligand diversity. We therefore generated chimeras between Activins (EGF-CFC-independent) and Sqt or Vg1 (EGF-CFC-dependent) (Figures 4 and 5). As a readout for active signaling, we injected mRNAs encoding these chimeric ligands into wild-type and MZo*ep* zebrafish embryos and analyzed the ectopic induction of the downstream genes *ntl* and *gsc*. Sqt, Vg1, and Activins induced these genes in wild-type embryos, allowing us to determine which chimeric ligands were active. Activins, but not Sqt or Vg1, were active in MZ*oep* mutants, allowing us to test which sequences conferred EGF-CFC coreceptor dependence or independence.

Initially, swaps of the Finger 1, Heel, or Finger 2 domains of Sqt and ActivinβB were generated. As shown in Figure 4, the Finger 2 region of ActivinβB contains sequence determinants that conferred EGF-CFC-independent activity on chimeric ligands. Chimeric SSA, ASA, and SAA that contain the Finger 2 region of ActivinβB were active in both wild-type and MZ*oep* embryos (Figure 4). To further delineate this region, we generated additional chimeras (Figure 5B). Short stretches of full-length Sqt were replaced by the corresponding region of ActivinβB, including the β6β7, loop, β8, or β9 subregions (Figure 5A and 5B; data not shown). Analysis of these chimeras revealed that the 14 amino acids encoding the loop and β8 region of ActivinβB (Sqt^Act^ ^β^ ^B[loop^ ^β^ ^8]^; the bracketed region in superscript denotes substituted domains) were sufficient to confer EGF-CFC independence. Further dissection of this region into loop alone (Sqt^Act^ ^β^ ^B[loop]^) or β8 alone (Sqt^Act^ ^β^ ^B[^ ^β^ ^8]^) yielded no or much weaker activity in MZ*oep* mutants as compared with wild-type embryos (Figure 5B and 5C). These results suggest that a 14 amino acid region in Activin is sufficient to confer EGF-CFC independence when placed into Sqt.

### Activin Loop-β8 Region Confers EGF-CFC Coreceptor Independence to Vg1

To determine whether the loop-β8 region has a wider role in conferring coreceptor independence, we generated additional chimeras using ActivinβA (another EGF-CFC-independent ligand) and Vg1. Sqt^Act^ ^β^ ^A[loop^ ^β^ ^8]^ (full-length Sqt with an ActivinβA loop-β8 region) and Vg1^Act^ ^β^ ^B[loop^ ^β^ ^8]^ (Vg1 with an ActivinβB loop-β8 region) both induced *gsc* expression in MZ*oep* mutants with similar efficiencies as in wild-type embryos (Figure 5D). These results were also corroborated using the A3-luc reporter assay (Figure 5E) and suggest that the loop-β8 region has a general role in conferring EGF-CFC coreceptor independence.

### Activin Loop-β8 Region Confers Binding to Activin Receptors in the Absence of EGF-CFC Coreceptors

Sqt^Act^ ^β^ ^B[loop^ ^β^ ^8]^ can signal in an EGF-CFC-independent manner in vivo, suggesting that this chimeric protein might bind to ActRIIB and Alk4 receptors in the absence of EGF-CFC coreceptors. To test this idea, we coexpressed and immunoprecipitated epitope-tagged ligands (ActivinβB/HA, Sqt/HA, Sqt^Act^ ^β^ ^B[loop^ ^β^ ^8]^/HA), receptors (ActRIIB[KR]/Myc and Alk4[KR]/Flag), and a coreceptor (Cripto/Flag) in *Xenopus* embryos (Yeo and Whitman 2001; Cheng et al. 2003). We found that Sqt binding to the ActRIIB and Alk4 receptor complex required Cripto (Figure 5F). In contrast, ActivinβB and Sqt^Act^ ^β^ ^B[loop^ ^β^ ^8]^ can bind to Activin receptors in the absence of Cripto. Moreover, Cripto did not significantly increase Sqt^Act^ ^β^ ^B[loop^ ^β^ ^8]^ ligand–receptor complex formation. These results indicate that the loop-β8 region is a determinant of TGFβ ligand binding to Activin receptors independent of EGF-CFC coreceptors.

### Multiple Residues in the Loop-β8 Region Contribute to Coreceptor Independence

**Figure 6:**
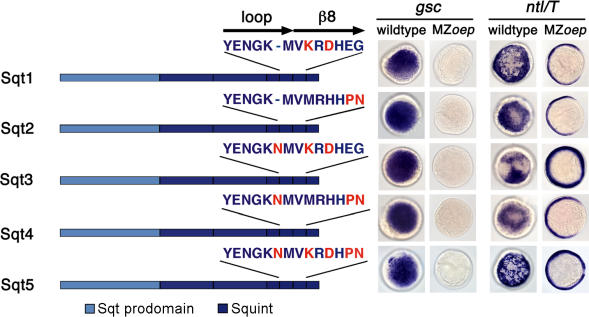
Conserved Residues in Activin Loop-β8 Region Confer Independence from EGF-CFC Coreceptors Synthetic mRNAs (200 pg) encoding Sqt harboring multiple mutations from ActivinβB (shown in red) were injected into wild-type and MZ*oep* embryos. *gsc* and *ntl* mRNA expression is shown at shield stage; animal pole views are dorsal to the right. Schematic is not drawn to scale. Note that the Sqt3 and Sqt5 constructs containing the Lys102–X–Asp104 motif and Asn99 insertion show weak expansion of *ntl* expression animally and dorsally in MZ*oep* mutants.

An alignment of the loop-β8 region of EGF-CFC-dependent and EGF-CFC-independent TGFβs (Figure 5A) reveals the presence of several residues unique to Activins. These include (i) a Lys102–X–Asp104 motif (numbering from ActivinβA) that forms a significant binding interface with the type II receptor ActRII (Wuytens et al. 1999; Greenwald et al. 2003; Thompson et al. 2003); (ii) Gln/Pro106 and Asn107, which contribute to the dimerization interface responsible for conformational arrangement (Thompson et al. 2003); and (iii) an Asn insertion at position 99. We therefore mutated the corresponding residues in Sqt, individually or in combination, to the ActivinβB sequence and tested them in the *gsc*/*ntl* induction assay (Figure 6). All constructs were active in wild-type embryos. The Sqt3 and Sqt5 constructs containing the Lys102–X–Asp104 motif and Asn99 insertion showed weak expansion of *ntl* expression animally and dorsally in MZ*oep* mutants. The incorporation of Pro106–Asn107 (Sqt2, Sqt4, and Sqt5) in Sqt did not enhance activity in MZ*oep* mutants. These results suggest that multiple residues contribute to coreceptor independence, with the type II receptor-binding interface being an essential determinant.

### The Loop-β8 Region in Sqt Is Inhibitory 

**Figure 7:**
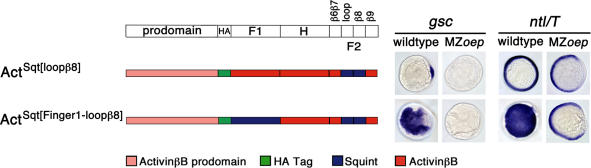
Sequence Determinants Conferring EGF-CFC Dependence Synthetic mRNAs (200 pg) encoding ActivinβB with single or double region substitutions from Sqt were injected into wild-type and MZ*oep* embryos. *gsc* and *ntl* mRNA expression is shown at shield stage; animal pole views are dorsal to the right. Schematic is not drawn to scale. HA indicates a hemagglutinin epitope tag. Note that Act^Sqt[loop^ ^β^ ^8]^ containing the loop-β8 region of Sqt is inactive in both wild-type and MZ*oep* embryos. In Act^Sqt[Finger1-loop^ ^β^ ^8]^, the additional substitution of Sqt Finger 1 region relieves the inhibitory presence of the Sqt loop-β8 region. Similar to Sqt, Act^Sqt[Finger1-loop^ ^β^ ^8]^ can induce ectopic *gsc* and *ntl* in wild-type, but not in MZ*oep* embryos. Western blot analysis indicates that these chimeric constructs produce stable ligands (data not shown).

The results described above identified the loop-β8 region of Activin as a region that confers coreceptor-independent signaling to ligands that are normally EGF-CFC-dependent. In a reverse set of experiments, we asked which regions confer dependence on EGF-CFC coreceptors. To identify domains that confer EGF-CFC dependence, chimeras between ActivinβB and Sqt (see Figures 4 and 7) were analyzed for their inability to signal in MZ*oep* mutants. Chimeras containing the Sqt Finger 2 domain (AAS and ASS; see Figure 4) or only the Sqt loop-β8 region (Act^Sqt[loop^ ^β^ ^8]^; Figure 7) were inactive in both wild-type and MZ*oep* embryos. Western blot analysis demonstrated that these chimeras generate stable ligands (data not shown). The addition of Finger 1 in SAS or Act^Sqt[Finger1-loop^ ^β^ ^8]^ relieved the inhibitory effect of the loop-β8 region of Sqt in wild-type embryos (Figure 7). These chimeras were inactive in MZ*oep* mutants. These results indicate that the loop-β8 region in Sqt acts as an inhibitory domain and that Finger 1 relieves this inhibition by conferring dependence on EGF-CFC coreceptors.

### Specificity of Antagonism by Lefty Is Determined by EGF-CFC Coreceptor Dependence

**Figure 8:**
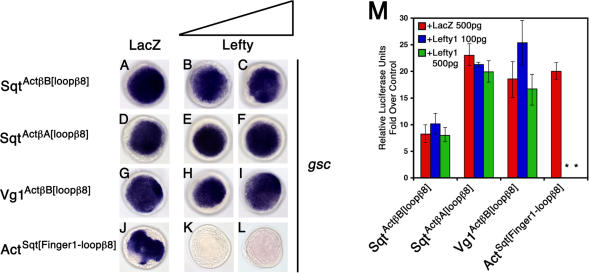
EGF-CFC Coreceptor Depen-dence Determines Susceptibility to Antagonism by Lefty (A–L) Embryos were injected with 75 pg of *Sqt^ActβB[loopβ8]^* mRNA (A–C), 75 pg of *Sqt^ActβA[**loop**β**8**]^* mRNA (D–F), 200 pg of *Vg1^ActβB[loopβ8]^* mRNA (G–I), or 200 pg *Act^Sqt[Finger1-loopβ8]^* mRNA (J–L). Embryos were further double-injected with either 500 pg of *LacZ* mRNA (A, D, G, and J), 100 pg of *lefty1* and 400 pg *LacZ* mRNAs (B, E, H, and K), or 500 pg of *lefty1* mRNA (C, F, I, and L). *gsc* mRNA expression in wild-type zebrafish embryos is shown at shield stage, animal pole view. Note that both levels of Lefty1 cannot inhibit the ectopic *gsc* expression induced by Sqt^Act^ ^β^ ^B[loop^ ^β^ ^8]^ (B and C), Sqt^Act^ ^β^ ^A[loop^ ^β^ ^8]^ (E and F), and Vg1^Act^ ^β^ ^B[loop^ ^β^ ^8]^ (H and I). In contrast, Lefty1 can inhibit Act^Sqt[Finger1-loop^ ^β^ ^8]^ (K and L). (M) Wild-type embryos were injected with 75 pg of either Sqt^Act^ ^β^ ^B[loop^ ^β^ ^8]^, Sqt^Act^ ^β^ ^A[loop^ ^β^ ^8]^, Vg1^Act^ ^β^ ^B[loop^ ^β^ ^8]^, or 200 pg of Act^Sqt[Finger1-loop^ ^β^ ^8]^ mRNA. Embryos were further double-injected with 500 pg of *LacZ* mRNA, 100 pg of *lefty1*, and 400 pg of *LacZ* mRNAs, or 500 pg of *lefty1* mRNA. Smad2 pathway activation was measured by an Activin response element luciferase reporter, A3-luc. Values are folds over wild-type control injected with 500 pg of *LacZ* mRNA and A3-luc reporter. An asterisk indicates a significant difference from the level of activation with ligand and *LacZ* expression alone (Student's *t*-test, *p* < 0.05).

Our genetic and biochemical studies suggested that Lefty blocks Nodal and Vg1 signaling via EGF-CFC coreceptors. In contrast, the coreceptor-independent signaling by Activins cannot be blocked by Lefty. This finding predicts that the EGF-CFC-independent chimeric ligands Sqt^Act^ ^β^ ^B[loop^ ^β^ ^8]^, Sqt^Act^ ^β^ ^A[loop^ ^β^ ^8]^, and Vg1^Act^ ^β^ ^B[loop^ ^β^ ^8]^ should also be resistant to Lefty. Conversely, the coreceptor-dependent chimera Act^Sqt[Finger1-loop^ ^β^ ^8]^ should be suspectible to inhibition by Lefty. To test this hypothesis, we coexpressed chimeric ligands and Lefty1 and analyzed *gsc* expression and A3-luc reporter induction (Figure 8A–8M). As predicted, Lefty1 did not inhibit signaling by Sqt^Act^ ^β^ ^B[loop^ ^β^ ^8]^ (Figure 8B and 8C), Sqt^Act^ ^β^ ^A[loop^ ^β^ ^8]^ (Figure 8E and 8F), or Vg1^Act^ ^β^ ^B[loop^ ^β^ ^8]^ (Figure 8H and 8I), but antagonized Act^Sqt[Finger1-loop^ ^β^ ^8]^ (Figure 8K and 8L). These results indicate that the incorporation of the Activin loop-β8 region into Nodal and Vg1 can render these ligands EGF-CFC-independent and therefore resistant to Lefty.

## Discussion

### Lefty Antagonizes EGF-CFC Coreceptors

**Figure 9:**
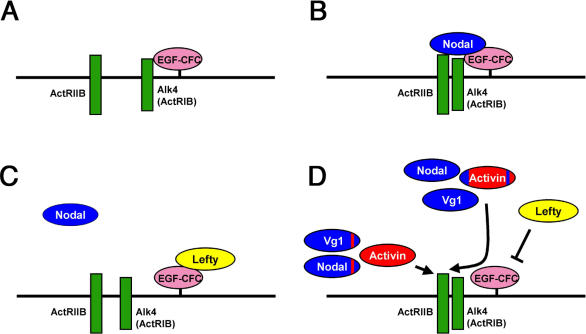
Model for EGF-CFC, Activin Receptors, Lefty, and TGFβ Interactions (A) In the absence of ligands, the EGF-CFC coreceptor (solid pink) is constitutively bound to the type I receptor Alk4 (solid green). (B) Nodal (solid blue) binds to receptor complexes consisting of EGF-CFC/Alk4 and ActRIIB (solid green). (C) Lefty (solid yellow) sequesters the EGF-CFC coreceptor, thereby preventing Nodal binding to the receptor complexes. (D) Subtle sequence differences determine the interaction with the EGF-CFC coreceptor and the Lefty inhibitor. Nodal and Vg1/GDF1 (solid blue) require the EGF-CFC coreceptor for signaling through ActRIIB and Alk4, while Activin (solid red) does not. Sqt^Act^ ^β^ ^B[loop^ ^β^ ^8]^ and Vg1^Act^ ^β^ ^B[loop^ ^β^ ^8]^ (solid blue with red strip) containing the loop-β8 region of ActivinβB can bind to ActRIIB and Alk4 without the EGF-CFC coreceptor and therefore cannot be blocked by Lefty. Act^Sqt[Finger1-loop^ ^β^ ^8]^ (solid red with two blue strips) requires the coreceptor for receptor complex binding and can be inhibited by Lefty.

Lefty molecules are key regulators of mesendoderm development and left–right axis determination, but the molecular basis of Lefty-mediated antagonism of Activin-like pathways has been elusive (Hamada et al. 2002; Schier 2003). Our genetic and biochemical studies provide three lines of evidence that Lefty blocks EGF-CFC coreceptors. First, Lefty only inhibits EGF-CFC-dependent TGFβ ligands such as Nodal and Vg1, but not EGF-CFC-independent ligands such as Activins. A striking example of this coreceptor-specific interaction is the finding that changing only 14 amino acids in Nodal or Vg1 to the corresponding residues in Activins renders the resulting TGFβ ligands independent of EGF-CFC coreceptors and resistant to Lefty. Second, the EGF-CFC proteins mouse Cripto and zebrafish Oep can partially suppress the effects of Lefty overexpression in zebrafish. Third, Leftys can bind to EGF-CFC coreceptors and block the coreceptors from interacting with Nodal. Furthermore, Lefty/EGF-CFC complexes seem to exclude interactions with type I and type II Activin receptors. Taken together, these results indicate that Lefty blocks a subset of TGFβ signals by the novel mechanism of blocking pathway-specific coreceptors (Figure 9A–9D).

The observation that Lefty does not block signaling by Activin seems in apparent contrast to previous studies that led to naming some Lefty family members Antivins, for their anti-Activin properties (Thisse and Thisse 1999; Cheng et al. 2000; Ishimaru et al. 2000; Tanegashima et al. 2000). In particular, it has been found that misexpression of Activin can suppress the defects caused by Lefty misexpression in vivo (Thisse and Thisse 1999). Our results do not undermine this finding, but suggest an alternative explanation. Previous studies have shown that Activin can suppress the loss of EGF-CFC activity in MZ*oep* mutants (Gritsman et al. 1999; Cheng et al. 2003). Analogously, we suggest that the blocking of EGF-CFC activity by Lefty can be bypassed by Activin, because this ligand can activate Activin receptors independent of co-receptors. A similar scenario can also account for the suppression of Lefty gain-of-function phenotypes by misexpression and activation of Activin receptors (Meno et al. 1999; Thisse and Thisse 1999; Sakuma et al. 2002). Hence, Activin and Activin receptors bypass the loss of EGF-CFC coreceptor function that is caused either by mutations in *oep* or by overexpression of Lefty. Conversely, Lefty cannot block Activin signals and Activin receptors because of their independence from EGF-CFC coreceptors.

Is the block of EGF-CFC coreceptors by Lefty a general and conserved mechanism? Although we have only analyzed a representative subset of these protein families (zebrafish and mouse Lefty1; zebrafish Oep; mouse Cripto; zebrafish Sqt), previous studies have suggested that heterologous Nodal, Lefty, and EGF-CFC proteins have similar activities in zebrafish (Schier 2003). For example, mouse Nodal, mouse Lefty2, and mouse Cripto are active in zebrafish, despite less than 30% overall sequence conservation with their zebrafish counterparts (Toyama et al. 1995; Meno et al. 1999; Gritsman et al. 1999). These studies suggest that the molecular mechanisms described here apply to most, if not all, Nodal/Lefty/EGF-CFC interactions. This does not exclude the possibility that Lefty has additional means of blocking TGFβ signaling. First, Leftys might block the processing of Nodals. However, Sqt is processed normally at levels of Lefty that block Nodal signaling (unpublished data). Second, Leftys might bind Nodal signals. This could result in blocking receptor interactions or antagonizing TGFβ dimerization. However, Sqt is not bound by Lefty at Lefty levels that block Nodal signaling and lead to complex formation with Cripto (unpublished data). Moreover, a Sqt protein containing the loop-β8 region of Activin is resistant to Lefty, whereas changing only the dimerization residues in this region does not confer resistance. Third, Leftys might interact with additional extracellular factors. Indeed, the overexpression of the extracellular domain of the type II receptor ActRIIB has been shown to suppress Lefty activity (Meno et al. 1999). Although zebrafish Lefty1 does not appear to bind to ActRIIB, it is conceivable that overexpression of soluble ActRIIB might protect EGF-CFC coreceptors or another yet-to-be identified protein from antagonism by Lefty. In addition, overexpression of EGF-CFC proteins in zebrafish does not induce dominant phenotypes (Gritsman et al. 1999). It is therefore possible that an additional factor would be required to completely block Lefty in these experiments. Alternatively, overexpression levels of EGF-CFC coreceptors might not be high enough to block Lefty at blastula stages. It is also possible that coreceptor overexpression might block Nodal signals, because it has been shown that EGF-CFC proteins and Nodals can directly interact. The complex feedback interactions between Lefty and Nodal might also overcome an initial reduction of Lefty activity by increasing Lefty transcription. These considerations and the data presented here therefore suggest that a major, but perhaps not exclusive, role of Leftys is to block a subset of TGFβ signals by interaction with EGF-CFC coreceptors.

### Implications for the Role of Lefty during Development

Our finding that Leftys can block Vg1 signaling also has important implications for the developmental control of TGFβ signaling. Based on previous studies revealing that Lefty proteins inhibit Nodal signaling, the mouse Lefty mutant phenotypes have been interpreted as a consequence of increased or sustained Nodal signaling (Hamada et al. 2002; Schier 2003). For example, loss of mouse Lefty2 has been thought to increase Nodal signaling, resulting in an enlarged primitive streak (Meno et al. 1999). Similarly, the left–right defects observed in mouse *Lefty1* and left-side specific *Lefty2* (*Lefty2^ΔASE^*) mutants have been thought to be caused by inappropriate spread of Nodal signaling (Meno et al. 1998, 2001). Our finding that Vg1/GDF1 signaling can also be blocked by Lefty suggests a more complex scenario. In particular, GDF1 (the mouse homologue of Vg1) is required for proper left–right axis determination (Rankin et al. 2000). *GDF1* mutants appear to have the opposite phenotypes as *Lefty1* and *Lefty2^ΔASE^* mutants. While GDF1 promotes the expression of left-side-specific genes such as *Pitx2* on the left, Leftys appear to block *Pitx2* expression on the right (Meno et al. 1998, 2001; Rankin et al. 2000). In light of our findings, we suggest that during left–right axis formation, Leftys act as antagonists of not only Nodal, but also GDF1. In this scenario, loss of Lefty1 or Lefty2 would lead to ectopic and sustained GDF1 signaling. This model is particularly attractive when one considers the expression patterns of *Lefty1*, *Lefty2*, the EGF-CFC gene *Cryptic*, *Nodal*, and *GDF1. Lefty 1* and *GDF1* are coexpressed in the developing midline (Meno et al. 1996, 1997; Rankin et al. 2000), whereas *Lefty2* and *Nodal* are coexpressed in left-lateral plate mesoderm (Conlon et al. 1994; Meno et al. 1997). *Cryptic* is expressed in both the lateral plate and midline (Shen et al. 1997). It is therefore conceivable that GDF1 signaling is restricted by Lefty1-mediated inhibition of Cryptic in the midline and its progenitors, whereas Nodal signaling is antagonized by Lefty2-mediated block of Cryptic in the lateral plate.

Our results might also have implications for the role of Cripto in tumorigenesis. Cripto is highly overexpressed in human epithelial cancers, such as breast and colon carcinomas (Salomon et al. 2000), and has been implicated in tumor formation (Ciardiello et al. 1991, 1994; Baldassarre et al. 1996; De Luca et al. 2000; Salomon et al. 2000; Adkins et al. 2003). Although the mechanisms by which Cripto acts in these circumstances are unclear, inhibition of Cripto by antisense or antibody blockade can inhibit tumor cell proliferation (Ciardiello et al. 1994; Baldassarre et al. 1996; De Luca et al. 2000; Adkins et al. 2003; reviewed by Shen 2003). Since Lefty is an in vivo antagonist of EGF-CFC activity, it might also serve as a therapeutic agent to block Cripto.

### Subtle Sequence Differences Determine the Interaction with Coreceptors and Inhibitors

The finding that the highly related ligands Activin, Nodal, and Vg1/GDF1 activate the same receptors but differ in their dependence on coreceptors allowed us to determine how ligand diversity and signaling specificity can be achieved. We have identified the loop-β8 region as a 14 amino acid domain, a mere 4% of the entire TGFβ signal, that contributes to coreceptor dependence or independence. Sqt and Vg1 incorporating the loop-β8 region of Activin can bind to the Activin receptors in the absence of EGF-CFC proteins. Conversely, Activin incorporating the loop-β8 region of Sqt is inactive, suggesting that the Nodal/Vg1 loop-β8 region might be inhibitory. This inhibition can be relieved by the Finger 1 domain of Sqt, which results in the dependence on EGF-CFC coreceptors (Figure 9D). These results indicate that rather subtle sequence variations can lead to striking changes in ligand diversity.

Structural considerations suggest that the loop-β8 region determines coreceptor independence or dependence at least in part by its interactions with type II receptors. The conserved Lys102–X–Asp104 motif in the Activin loop-β8 region has been shown to be important for high-affinity binding to the ActRII receptor (Wuytens et al. 1999; Greenwald et al. 2003; Thompson et al. 2003). In the crystal structure of the ActivinβA-ActRIIB complex, Lys102–X–Asp104 forms an intramolecular salt bridge that interacts with a hydrophobic interface on ActRIIB (Thompson et al. 2003). Mutational analysis has shown that substituting Lys102 with a neutral charge (Ala) significantly reduces receptor binding affinity and signaling (Wuytens et al. 1999). In contrast to Activin, EGF-CFC-dependent ligands such as Nodals and Vg1/GDF1 have the differentially charged residues Met/Leu102 and His104 at the corresponding positions (numbering according to ActivinβA). Similarly, in BMP7 the corresponding residues are Leu102 and Lys104. It has been shown that modeling Lys onto the aligned 102 residue in BMP7 positions it within hydrogen-bonding distance to Glu29 of ActRII and may allow for greater hydrophobic packing at the interface (Greenwald et al. 2003). Analogously, we propose that Sqt^Act^ ^β^ ^A[loop^ ^β^ ^8]^, Sqt^Act^ ^β^ ^B[loop^ ^β^ ^8]^,Vg1^Act^ ^β^ ^B[loop^ ^β^ ^8]^, Sqt3, and Sqt5 are coreceptor-independent because of their favorable binding to ActRIIB receptors. Conversely, the corresponding region in Sqt and Vg1 might be inhibitory because of inefficient interaction with ActRIIB receptors. Detailed structural studies should reveal whether EGF-CFC proteins overcome this inhibition by changing the conformation of Nodal and Vg1 or by providing an additional interaction surface that allows the assembly of receptor complexes.

In summary, our results lead to two major conclusions. First, Lefty inhibits a subset of TGFβ signals by using the novel mechanism of blocking pathway-specific coreceptors belonging to the EGF-CFC family. Second, subtle sequence changes in TGFβs determine their signaling specificity and dependence on coreceptors. Although *Drosophila* has an Activin signaling pathway, Nodals, Leftys, and EGF-CFC proteins seem to be restricted to chordates (Brummel et al. 1999; Schier 2003). The evolution of Activin-like signaling pathways therefore represents a remarkable example of how a simple signaling pathway consisting of ligand and receptors can be diversified by subtle sequence changes that modulate the interaction with coreceptors and their inhibitors.

## Materials and Methods

### 

#### Strains and embryos.

Adult homozygous fish for *oep^tz57^* were generated as described previously (Zhang et al. 1998; Gritsman et al. 1999). *Xenopus* embryos were obtained as described in Hemmati-Brivanlou et al. (1992).

#### Generation of constructs.

Epitope-tagged and chimeric constructs were made using PCR-based methods and confirmed by sequencing. pCS2-zebrafish Lefty1/HA and Lefty1/Glu constructs were generated by inserting three tandem copies of HA-epitope or Glu-epitope, respectively, after Val145. The initial three-lettered (XXX) Sqt/ActivinβB chimeras were generated by subcloning the prodomain of *Xenopus* ActivinβB (codons Met1 to Gly256) fused to an HA-epitope/XhoI fragment (YPYDVPDYALE) and followed by the mature chimeric ligand into pcDNA3 vector. *S* denotes Sqt; *A* denotes ActivinβB. The boundaries for Sqt mature ligand domains are as indicated: Finger 1 (Asn263 to Cys325), Heel (Pro326 to Cys358), and Finger 2 (Val359 to His392). The boundaries for *Xenopus* ActivinβB mature ligand domains are as indicated: Finger 1 (Cys215 to Cys299), Heel (Pro300 to Cys335), and Finger 2 (Ile336 to Ala370). Full-length chimeras were generated by incorporating the indicated regions into Sqt, ActivinβB, or zebrafish Vg1, which were then subcloned into the pT_7_T_S_ vector (Ekker et al. 1995). The boundaries for Sqt Finger 2 structural subregions are as indicated: β6β7 (Val359 to Try370), loop (Tyr371 to Met376), β8 (Val377 to Gly383), and β9 (Met384 to His392). The boundaries for *Xenopus* ActivinβB Finger 2 structural subregions are as indicated: β6β7 (Ile336 to Try347), loop (Phe348 to Ile354), β8 (Val355 to Asn356), and β9 (Met357 to Ala370). The *Xenopus* ActivinβA loop-β8 region sequence is FDRNNNVLKTDIAD (also identical in *Xenopus* ActivinβD). The zebrafish Vg1 loop-β8 region is from Try332 to Asp345. pcDNA3-zebrafish Vg1/HA, pcDNA3-Squint/HA, pCS2-Alk4(KR)/Flag (a kinase-defective mutant of human Alk4 with Lys234 to Arg234 substitution), pCS2-ActRIIB(KR)/Myc (a kinase-defective mutant of mouse ActRIIB with Lys217 to Arg217 substitution), and pCS2-Cripto/Flag have been described elsewhere (Yeo and Whitman 2001; Cheng et al. 2003).

#### Embryo microinjection.

Plasmids were linearized and sense strand-capped mRNA was synthesized using the mMESSAGE mMACHINE system (Ambion, Austin, Texas, United States). Zebrafish embryos were dechorinated by pronase treatment and injected between the one- and four-cell stage. *Xenopus* embryos at the one- to two-cell stage were used for injections into the animal pole.

#### Phenotypic analysis.

Zebrafish embryos at 24 h were mounted in 2% methylcellulose and photographed using a Zeiss (Oberkochen, Germany) M2Bio dissecting microscope. In situ hybrization was performed as described previously (Thisse et al. 1993), using RNA probes to *gsc* and *ntl* (Stachel et al. 1993; Schulte-Merker et al. 1994).

#### Luciferase reporter assay.

Luciferase assays were performed with three to six samples and five embryos in each sample. Results are representative of three independent experiments. The injection mixtures were equalized with respect to total mRNA amount with *LacZ* mRNA. The A3-luc reporter DNA construct (25 pg) (Chen et al. 1996) was also coinjected. Whole zebrafish embryos were harvested at shield stage. Luciferase activity was analyzed using the Luciferase Reporter Assay system (Promega, Madison, Wisconsin, United States) according to the manufacturer's instruction in a Lumat LB9501 (Berthold Technologies, Bad Wildbad, Germany). Owing to the technical aspects of microinjections, in rare circumstances, a single outlier was statistically removed from a population using Grubbs' test/extreme studentized deviate method. Inclusion of outliers into the populations does not change the statistical significance of the *p* values; that is, *p* remains <0.05, where indicated.

#### Coimmunoprecipitation analysis.

*Xenopus* embryos were harvested at stage 10. For chemical cross-linking of proteins, animal halves were incubated in PBS with 10 mM 3,3′-dithiobis(sulfo-succinimidyl propionate) (DTSSP) (Pierce Biotechnology, Rockford, Illinois, United States) and incubated for 2 h on ice. Coimmunoprecipitation was performed as described previously (Yeo and Whitman 2001). Purified processed mouse Lefty1, soluble mouse Cripto, and mouse VEGF-D proteins were obtained from R&D Systems (Minneapolis, Minnesota, United States). Activity assays were performed by R&D Systems. The proteins were incubated in PBS with 1 mM DTSSP for 1 h on ice. Coimmunoprecipitation was performed as described previously (Yeo and Whitman 2001). Samples were treated with 100 mM DTT to cleave DTSSP prior to SDS-PAGE analysis. The following antibodies were used for immunoprecipitation and Western blot analysis: anti-Flag mouse monoclonal antibody (clone M2; Sigma, St. Louis, Missouri, United States), anti-HA mouse monoclonal antibody (clone 16B12; Covance, Princeton, New Jersey, United States), anti-HA rabbit polyclonal antibody (Y-11; Santa Cruz Biotechnology, Santa Cruz, California, United States), anti-c-Myc rabbit polyclonal antibody (A-14; Santa Cruz Biotechnology), anti-c-Myc mouse monoclonal antibody (clone Ab-1; Oncogene Science, Tarrytown, New York, United States), anti-His mouse monoclonal antibody (clone 6-His; Covance), anti-mLefty1 goat polyclonal antibody (R&D Systems), and anti-Glu mouse monoclonal antibody (clone Glu-Glu; Covance). Proteins were visualized using the Super Signal West Pico/Femto Chemiluminescent Substrate system (Pierce).

## Supporting Information

### Accession Numbers

The GenBank (http://www.ncbi.nlm.nih.gov/entrez/query.fc-gi?db=Nucleotide) accession numbers for the sequences discussed in this paper are ActivinβA (Q9W6I6), ActivinβB (Q91350), human ActRIIB (P08476), mouse ActRIIB (P27040), rat ActRIIB (P38445), Alk4 (Z22536), BMP7 (P23359), Cripto (P51865), Cryptic (P97766), *cyclops* (P87358), GDF1 (P20863), *goosecoid* (P53544), Lefty1 (Q9W6I6), Lefty2 (P57785), mLefty1 (Q64280), Nodal (P43021), *no tail* (Q07998), *one-eyed pinhead* (O57516), *Pitx2* (P97474), *squint* (O13144), VEGF-D (P97946), and Vg1 (P09534).

## Abbreviations

ActβA: ActivinβA
ActβB: ActivinβB
ActRIIB: Activin receptor type IIB
Alk4: Activin receptor-like kinase-4
DTSSP: 3,3′-dithiobis(sulfo-succinimidyl propionate)
EGF-CFC: epidermal growth factor–Cripto/FRL-1/Cryptic
GDF1: growth and differentiation factor-1
*gsc*: *goosecoid*
GPI: glycosylphosphatidylinositol
HA: hemagglutinin
hpf: hours postfertilization
kDa: kilodalton
*LacZ*: E. coli β-galactosidease
luc: luciferase
MAPK: mitogen-activated protein kinase
MZ*oep*: maternal- and zygotic-deficient mutant of *oep*
*ntl*: *no tail*
*oep*: *one-eyed pinhead*
*sqt*: *squint*
TGFβ: transforming growth factor-β
VEGF-D: vascular endothelial growth factor-D
